# Supplementary figures and images for: Prior exposure to pathogens augments host heterogeneity in susceptibility and has key epidemiological consequences
Source: PLoS Pathog. 2024 Sep 4;20(9):e1012092. doi: 10.1371/journal.ppat.1012092 (PMC11404847; doi:10.1371/journal.ppat.1012092)

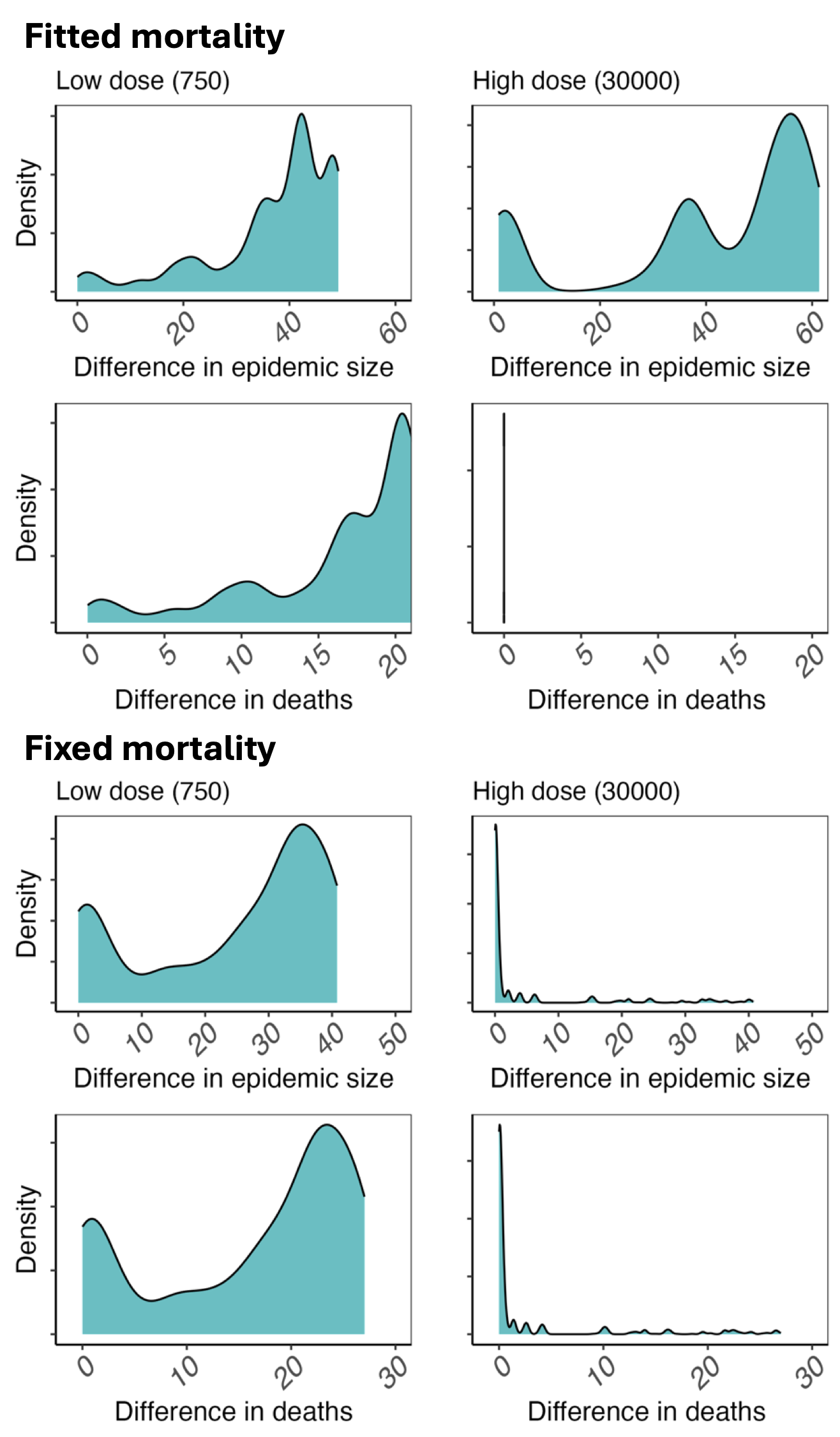

Supplement: S1 Fig — Density plots of observed increases (difference = homogeneous—heterogeneous value) in both epidemic size (left) and total mortality (right) for simulations using parameter estimates obtained from bootstrapping the chi-squared residuals of the dose-response parameters (Fig 3). Paired comparisons were made while holding mean susceptibility constant, and only varying whether susceptibility distributions were homogeneous versus heterogeneous (fitted parameters from each bootstrap sample). The top four panels are from simulations using mortality rate parameters fitted to empirical data for each prior exposure treatment; bottom four panels are from simulations using a fixed mortality rate (that of the no prior exposure group) across all prior exposure treatments. Density plots are scaled to the maximum height across a fixed x-axis scale (for fitted mortality, maximum of 60 for epidemic size and maximum of 20 for deaths; for fixed mortality, maximum of 50 for epidemic size and maximum of 30 for deaths). Confidence intervals for epidemic size differences are reported in the main text (see Results). For differences in deaths (hom-het), 95% CIs were: low dose, fitted mortality [0.9906,23.74]; high dose, fitted mortality [0,0]; low dose, fixed mortality [0.07863,27.01]; and high dose, fixed mortality [0.0064,24.09]. (TIFF) [file ppat.1012092.s001.tiff]
